# Repeated Courses of Radiosurgery for New Brain Metastases to Defer Whole Brain Radiotherapy: Feasibility and Outcome With Validation of the New Prognostic Metric Brain Metastasis Velocity

**DOI:** 10.3389/fonc.2018.00551

**Published:** 2018-11-22

**Authors:** Corinna Fritz, Kim Borsky, Luisa S. Stark, S. Tanadini-Lang, Stephanie G. C. Kroeze, Jérôme Krayenbühl, Matthias Guckenberger, Nicolaus Andratschke

**Affiliations:** Department of Radiation Oncology, University Hospital Zurich Zurich, Switzerland

**Keywords:** brain metastases (BM), stereotactic radiosurgery, repeat radiosurgery, brain metastasis velocity, whole brain radiation therapy (WBRT), salvage radiation therapy (SRT)

## Abstract

**Purpose:** Stereotactic radiosurgery (SRS) is the preferred primary treatment option for patients with a limited number of asymptomatic brain metastases. In case of relapse after initial SRS the optimal salvage treatment is not well defined. Within this retrospective analysis, we investigated the feasibility of repeated courses of SRS to defer Whole-Brain Radiation Therapy (WBRT) and aimed to derive prognostic factors for patient selection.

**Materials and Methods:** From 2014 until 2017, 42 patients with 197 brain metastases have been treated with multiple courses of SRS at our institution. Treatment was delivered as single fraction (18 or 20 Gy) or hypo-fractionated (6 fractions with 5 Gy) radiosurgery. Regular follow-up included clinical examination and contrast-enhanced cMRI at 3–4 months' intervals. Besides clinical and treatment related factors, brain metastasis velocity (BMV) as a newly described clinical prognostic metric was included and calculated between first and second treatment.

**Results:** A median number of 1 lesion (range: 1–13) per course and a median of 2 courses (range: 2–6) per patient were administered resulting in a median of 4 (range: 2–14) metastases treated over time per patient. The median interval between SRS courses was 5.8 months (range: 0.9–35 months). With a median follow-up of 17.4 months (range: 4.6–45.5 months) after the first course of treatment, a local control rate of 84% was observed after 1 year and 67% after 2 years. Median time to out-of-field-brain-failure (OOFBF) was 7 months (95%CI 4–8 months). WBRT as a salvage treatment was eventually required in 7 patients (16.6%). Median overall survival (OS) has not been reached. Grouped by ds-GPA (≤ 2 vs. >2) the survival curves showed a significant split (*p* = 0.039). OS differed also significantly between BMV-risk groups when grouped into low vs. intermediate/high risk groups (*p* = 0.025). No grade 4 or 5 acute or late toxicity was observed.

**Conclusion:** In selected patients with relapse after SRS for brain metastases, repeat courses of SRS were safe and minimized the need for rescue WBRT. The innovative, yet easy to calculate metric BMV may facilitate treatment decisions as a prognostic factor for OS.

## Introduction

Treatment of brain metastases is still a challenge since different treatment goals need to be weighed: for one thing preventing progression of metastases with its associated neurological deterioration then again ensuring quality of life and limiting treatment-associated morbidity (1). In the past, whole-brain radiation therapy (WBRT) was standard of care for symptom control and presumably prolonged survival. Still, with its risk of neurotoxicity and early functional impairment (2–5), its inferior rate of local control (6) and its questionable impact on overall survival (7) the role of WBRT in the treatment of limited numbers of brain metastases (≤4) has been challenged and transformed over the past 10 years.

Brain metastases serve as the ideal target for stereotactic radiosurgery (SRS) (1, 8) and the efficacy of this treatment has been proven in several prospective phase III trials (2, 4, 6, 9). Consequently, SRS alone has been recommended within national guidelines as the preferred radiation treatment option for up to four asymptomatic brain metastases (1, 10, 11) to defer WBRT. For patients with more than 4 metastases SRS without WBRT has equally shown encouraging results regarding overall survival and toxicity (12).

Despite the excellent local control rates of SRS only, up to 50% of patients will present with new brain metastases within a year in terms of distant brain failure (4, 6, 9, 12). The optimal salvage treatment in this situation has not yet been defined. WBRT is still administered, but challenges to WBRT remain the same as in the primary situation. Furthermore, the concept of salvage WBRT has been investigated particularly as a subset of patients with long term WBRT-free survival can be identified by predictive parameters (13). Therefore, repeat radiosurgery for new brain metastases to defer or even avoid WBRT represents an attractive concept, although data considering efficacy, safety or optimal patient selection is still missing. Only few groups have recently reported retrospective analyses of their experience with repeated courses of radiosurgery (14–18) and hence optimal patient selection appears crucial.

The graded prognostic assessment (GPA) predicts survival upon the initial diagnosis of brain metastases (19–22) and potentially remains prognostic for new lesions treated with repeated courses of SRS (23). Recently, Farris et al. have introduced a novel metric, prognostic of overall survival: the clinical metric brain metastasis velocity (BMV) serves as an estimate for development of new metastases over time and is not only associated with overall survival, but also predicts need of salvage therapy in case of distant brain failure or risk of neurological death (24).

Within this retrospective study, we present our results of the feasibility, toxicity, and outcome of repeated courses of radiosurgery for the treatment of new brain metastases and examine the value of the metric BMV.

## Patients and methods

### Patient eligibility

From February 2014 until August 2017, 42 patients with 197 brain metastases were treated with at least 2 courses of SRS for either intact brain lesions or resection cavities at the University Hospital of Zurich. According to institutional standards, repeat SRS was given, if all new lesions (≤ 9 lesions at a time) were deemed amenable for SRS (criteria defined in treatment section) and the patient was assessed in good performance status by the treating physician. A multi-disciplinary tumor board approved all indications.

All patients received a physical examination and an oncological re-staging by either computed tomography (CT) imaging or combined whole body positron emission tomography (PET) with CT imaging. Cerebral metastases were assessed by contrast enhanced high-resolution cranial magnetic resonance imaging (MRI).

### Treatment

According to institutional guidelines following international recommendations (1, 9, 12, 25, 26) treatment was delivered as follows:

All patients were immobilized using a dedicated frame-less stereotactic mask system (CIVCO Medical Solutions, Coralville, IA, USA). Planning-CT was done in treatment position using a 0.75–1 mm slice thickness and intravenous (IV) contrast agent. A dedicated planning MRI with IV contrast agent including a volumetric T1 sequence and a slice thickness of 0.6 mm was acquired. Both image modalities were fused using rigid image registration for target delineation. For contouring, the MRI-visible gross tumor volume (GTV) was delineated without additional margin as clinical target volume (CTV). The planning target volume (PTV) was derived by adding an isotropic margin of 1 mm. In contrast, for resected brain metastases the cavities were delineated as CTV. In this case, the PTV was derived by adding an isotropic margin of 2 mm. Patients were grouped for dose prescription: For 1–4 small metastases with diameter of ≤ 2.5 cm each, a single fraction scheme was used (1 × 18–20 Gy). If metastases measured > 2.5 cm, 6 fractions of 5 Gy were prescribed. For patients with 5–9 metastases, the threshold was a diameter of 1 cm for either single doses SRS or, if larger in size, 6 fractions of 5 Gy was used. If >9 metastases were treated, the regimen with 6 fractions was also favored. Postsurgical treatments were delivered in 6 × 5 Gy. All doses were prescribed to the PTV encompassing the 80%-isodose line and delivered as Volumetric Modulated Arc Therapy (VMAT) using True Beam or Edge linear accelerators (LINACs) (Varian Medical Systems, Inc., Palo Alto, CA, USA).

### Toxicity and endpoint definitions

We followed patients from time of initial SRS until death or closure of data-entry. For the first year, cMRI and clinical examination were appointed every 3 months and thereafter at 4 months' intervals. Toxicity was graded according to National Cancer Institute Common Terminology Criteria for Adverse Events (CTCAE v4.0) and classified as acute (up to 3 months after respective course of SRS) or late toxicity.

Treatment response and radiation necrosis (RN) as an adverse event were investigated following recommendations of the response assessment in neuro- oncology (RANO)-group (27). Gadolinium-enhanced MRI served as a basis for response assessment. Local control (LC) of a treated lesion was defined as either response or stable disease in the last follow-up cMRI scans. Newly diagnosed out-of-field lesions were regarded as out of field brain failure (OOFBF). In order to distinguish between true progression and pseudo-progression, time course over all follow-up MRI-scans was taken in account (28). The diagnosis of RN followed the RANO and was derived by suggested multimodal diagnostics (29): if RN was suspected in standard cMRI, susceptibility-weighted contrast enhanced (DSC) perfusion MRI or 18F-Fluorethyltyrosin (^18^FLT)-PET were performed. A RN was called symptomatic RN if a patient presented with clinical symptoms as nausea, headache or fatigue.

Dying with increased or new neurological dysfunction or progressive metastases was considered neurological death, whereas the status of stable metastases was not (30).

### Statistics

Actuarial survival time and tumor control as freedom from local failure (LF) or OOFBF were calculated according to the Kaplan-Meier method. OOFBF was assessed patient-specifically after the individual courses. In contrast, freedom from LF was determined as lesion-specific local control from time of corresponding course to date of the event. Overall survival was calculated from each course of SRS to the time of death.

To analyze prognostic factors for OS a Cox proportional hazard model (*P* < 0.05) was trained. Primarily a univariate analysis was performed, followed by a multivariate analysis of factors that were prognostic on univariate level. Histology (NSCLC vs. others) and diagnostic-specific GPA (0–2 vs. 2.5–4) were analyzed as categorical variables.

BMV was calculated as the cumulative number of new brain metastases that developed since initial SRS over time. Within our cohort it was determined at time of first DBF after the first course of SRS treatment. According to proposed risk profile groups (24), patients were classified as follows:

- low risk: < 4 new metastases/year.
- intermediate risk: 4–13 new metastases/year.
- high risk: >13 new metastases/year.

## Results

### Patient, tumor, and treatment characteristics

We identified 42 consecutive patients who received repeated courses of SRS from our institutional database at the University-Hospital Zurich. At time of first treatment most presented with good Karnofsky performance status (KPS) of median of 80% (range: 50–100%) and a median age of 56 years (range: 35–83 years). Ds-GPA was calculated individually with a median of 2.5 (range: 1–4). 12 of 23 patients (52%) presented with non-small cellular lung carcinoma as primary tumor diagnosis; 9 of these with adenocarcinoma. Further patient characteristics are summarized in Table 1.

**Table 1:** Baseline patient characteristics.

**Characteristics**	***n* (%)**
Total	42
**GENDER**	
Male	20 (48)
Female	22 (52)
AGE (Y)^*^	
Median (range)	59 (35–84)
**HISTOLOGY**	
Non-small cell lung carcinoma	20 (48)
Melanoma	7 (17)
Breast	6 (14)
Other	9 (21)
KPS (%)^*^	
< 70	1 (2)
70–80	16 (38)
90–100	25 (60)
Ds-GPA^*^	
Median (range)	2 (1–4)
**PATIENTS PER COURSE**	
Course no. 1	42 (100)
Course no. 2	42 (100)
Course no. 3	10 (24)
Course no. 4	3 (7)
Course no. 5	2 (5)
Course no. 6	1 (2)

* *At time of first treatment. Values are numbers (percentage) except where noted otherwise*.

*Ds-GPA, Diagnostic-specific graded prognostic assessment score; KPS, Karnofsky performance status*.

Within the observation period, a total number of 197 metastases from 100 SRS courses was treated. The median size of the GTV was 0.3 cm^3^ (range: 0.1–39.4 cm^3^) whereas the median size of the PTV was 0.7 cm^3^ (range: 0.1–55.4 cm^3^). The sum of all GTVs for a single patient at one treatment period was defined as aggregated volume (AV). The median AV per course was 1.2 cm^3^ (range: 0.1–48.4 cm^3^). Hundred and Twenty-One (61%) of the lesions were treated with a single dose SRS of 18 or 20 Gy. The remaining 76 lesions received fractionated treatments of 6 fractions with 5 Gy single dose (see treatment characteristics in Table 2).

**Table 2:** Lesion and treatment characteristics.

**Characteristics**	***n* (%)**
Total lesions	197
SRS target^*^	121 (61)
FSRT target	76 (39)
Intact metastasis	49 (25)
Post-resection cavity	27 (14)
**VOLUME GTV (cm**^3^**)**	
Median (range)	0.3 (0.1–39.4)
Median volume SRS lesions (range)	0.1 (0.1–8.2)
Median volume FSRT lesions (range)	2.45 (0.1–39.4)
**VOLUME PTV (cm**^3^**)**	
Median PTV overall (range)	0.7 (0.1–55.4)
**AGGREGATED VOLUME (AV) PER COURSE p.P. (cm**^3^**)**	
Median AV p.P. over all courses	1.2 (0.1–48.4)
**DOSE PRESCRIPTION**	
1 × 18 Gy	15 (8)
1 × 20 Gy	106 (54)
6 × 5 Gy	76 (38)
SRS/FSRT prescription isodose line	80%

*Values are numbers (percentage) except where noted otherwise*.

*SRS, Stereotactic radiosurgery; FSRT, fractionated stereotactic radiotherapy; p.P, per patient*.

* *With SRS, only intact metastasis and no post-resection cavities were treated*.

### Ds-GPA score

**Figure 1:**
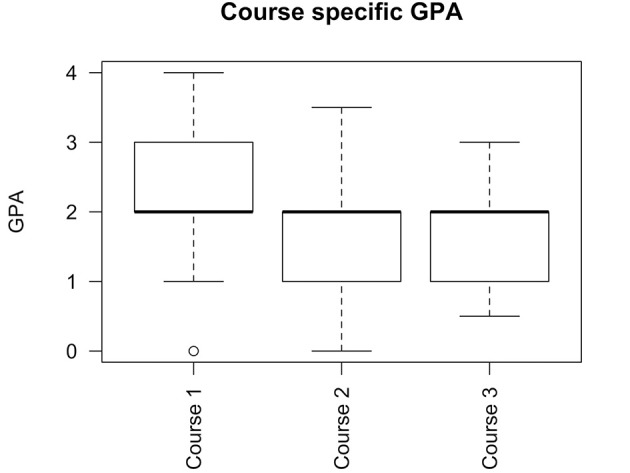
Course specific GPA. Boxplot showing median, 1st and 3rd quartile and 95% CI. A median GPA of 2 for all courses was observed.

As Figure 1 demonstrates the median ds-GPA remained stable during the entire treatment time. However, a shift of the quartiles and the range toward lower ds-GPA values was obvious within subsequent treatment courses.

### Brain metastasis velocity

Median 3.5 new metastases / year (range: 0.3–22.1) were observed at time of first distant failure. Twenty-Three of Forty-Two patients (55%) could be grouped within low-risk (< 4 new metastases/year), Twelve (28%) had an intermediate-risk (4–13 new metastases/year), and Seven of Forty-patients (17%) were in the high-risk group (>13 new metastases/year).

### Brain tumor control

**Figure 2:**
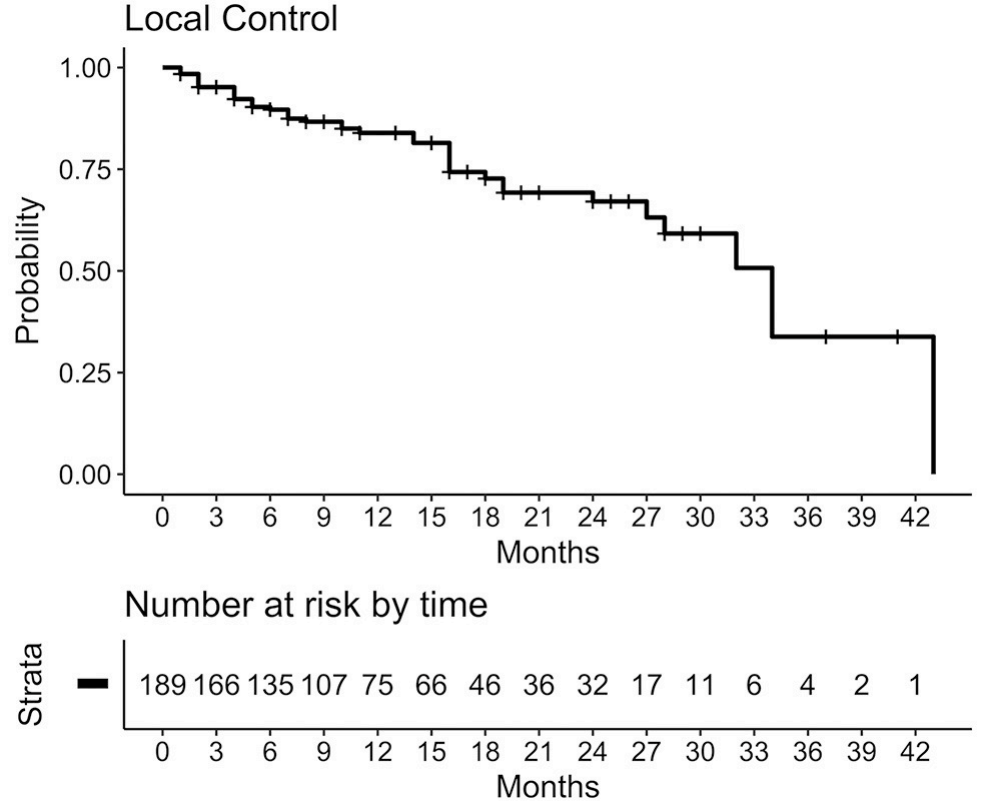
Kaplan-Meier-Plot for local control. For 189 lesions (with at least one follow-up with cMRI) a 1-year local control rate of 84% was observed, calculated from time of corresponding course to date of the event. Censored in case no event occurred or if WBRT had to be administered.

With a median follow-up of 17.4 months (range: 4.6–45.5 months) after the first course of treatment a local control rate of 84% was observed at 1 year and 67% at 2 years independently of treatment course. Figure 2 shows Kaplan-Meier-Plot for local control. There was no statistically significant difference to be found between lesions treated with SRS vs. fSRT (*p* = 0.6).

**Figure 3:**
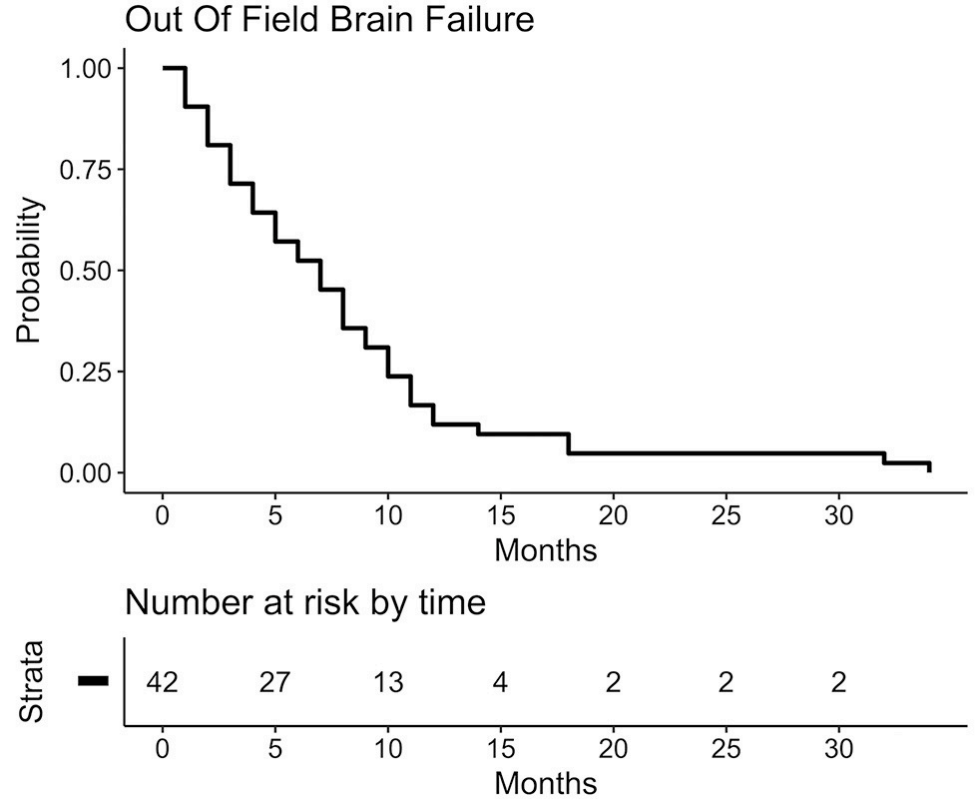
Kaplan-Meier-Plot for out-of-field brain control after 1st treatment course. Censored in case no event occurred or if WBRT had to be administered.

Considering overall brain control, median time to out-of-field-brain-failure (OOFBF) was 7 months (95%CI 4–8 months) after the first treatment and 6 months (95%CI 3–17 months) after the second. One-year and Two-year cumulative incidence of OOFBF after first course was 83 and 95%, respectively. Figure 3 shows Kaplan-Meier-Plot for OOFBF.

### Salvage WBRT

Seven of Forty-Two (16.6%) patients were not amenable to another course of repeat SRS at time of second recurrence and had received WBRT within a median interval of 6 months (range: 4–13 months) to the first SRS course.

### Patient survival: univariate and multivariate analysis

**Figure 4:**
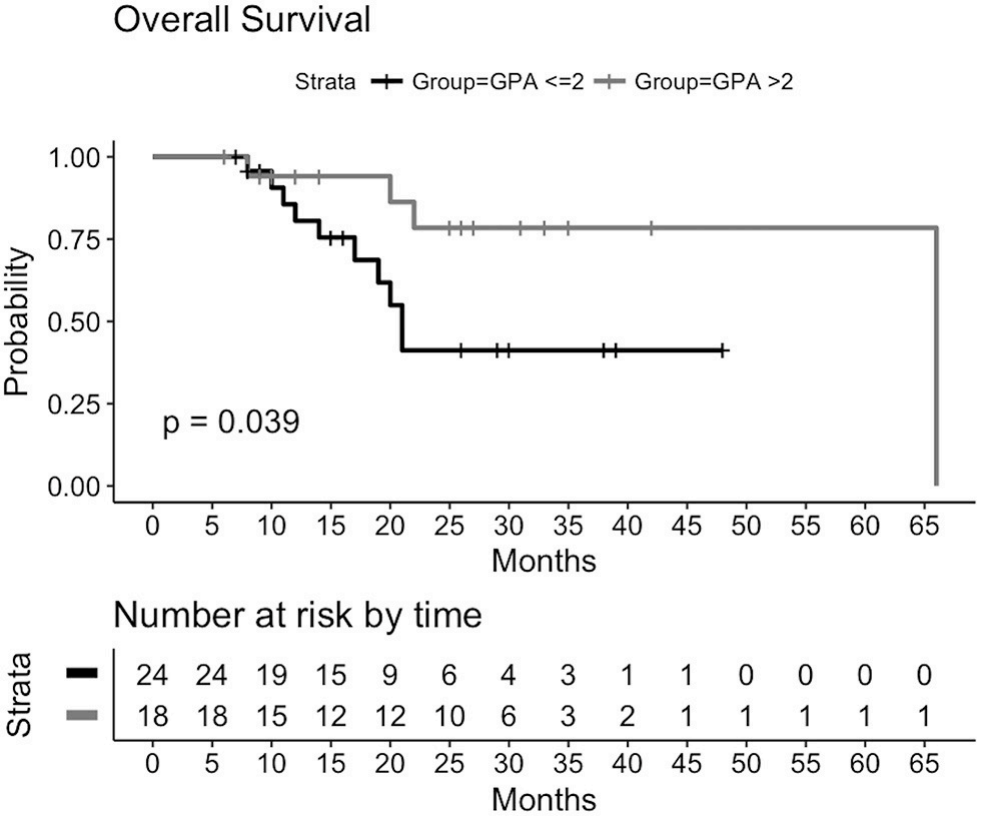
Kaplan-Meier-Plot for overall survival grouped by ds-GPA ≤ 2 vs. >2 after time of first diagnosis of brain metastases. Median overall survival for ds-GPA >2 after diagnosis of first brain metastasis has not been reached. For ds-GPA ≤ 2: median OS of 21 months (95% CI: 17-NA).

From date of first diagnosis of brain metastases a median OS has not been reached at time of final analysis. As Figure 4 demonstrates, the survival curves showed a significant split (*p* = 0.039), when grouped by ds-GPA (≤ 2 vs. >2). The median OS for patients with a ds-GPA >2 after diagnosis of first brain metastasis has not been achieved. A median OS of 21 months (95% CI: 17-NA) for patients with ds-GPA ≤ 2 was observed.

**Figure 5:**
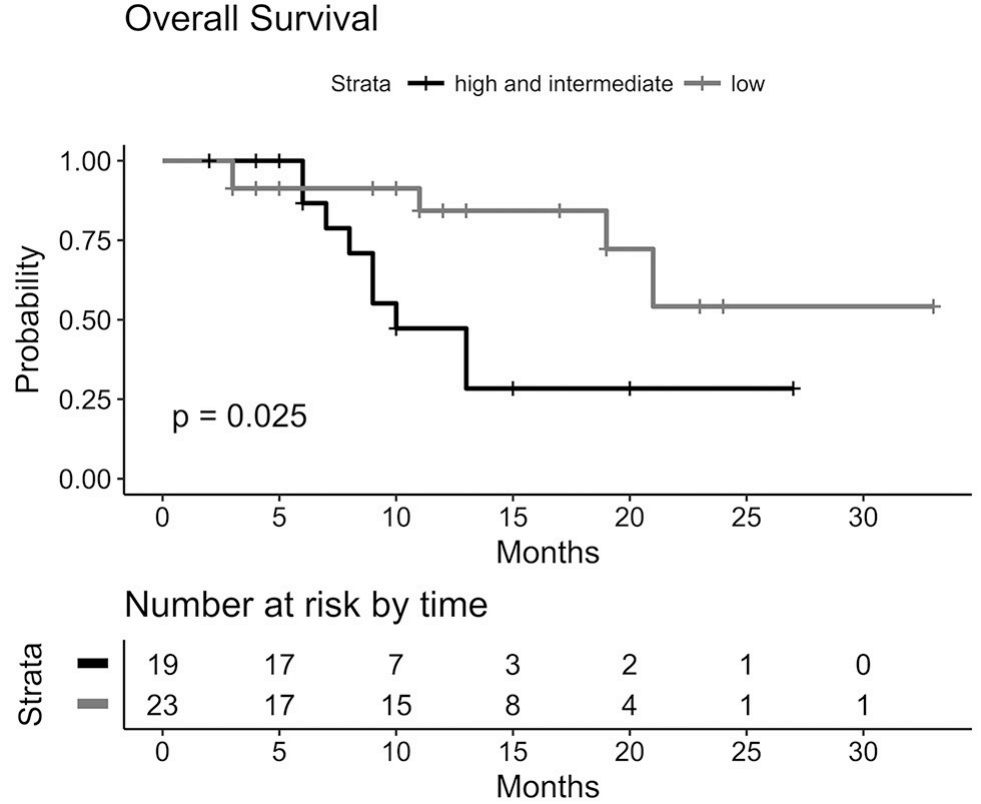
Kaplan-Meier-Plot for Overall Survival grouped by BMV risk group. For low-risk group median OS has not been reached after first DBF. Combined intermediate /high-risk group: median OS of 10 months (95%CI: 7-NA).

BMV proved to be a prognostic metric for OS calculated from time of first distant-brain-failure. Figure 5 displays how survival curves differ significantly for low-risk (23 patients, 55%) vs. intermediate- and high-risk patients (19 patients, 45%) (*p* = 0.025). Median OS from time of first DBF for the low-risk group has not been reached, whereas the median OS for the combined group was 10 months (95% CI: 7-NA).

At time of last follow-up 28 of 42 (67%) patients were still alive. Eight of Fourteen (57%) deaths can be attributed to neurological death according to the definition in the methods section.

Results of univariate analysis are shown in Table 3. On univariate analysis, local control of the primary tumor at time of second course and the calculated BMV after the first course correlated significantly with OS. For multivariate analysis we looked at the influence of ds-GPA, which already includes various clinical parameters, and additional non-included clinical parameters like aggregated treated tumor and brain metastases velocity. Results are shown in Table 4. BMV remained significantly correlating with OS [*p* = 0.04; HR: 1.10 (1.00–1.21)].

**Table 3:** Univariate analysis of correlation to overall survival course-specifically.

	**Variable**	***P***	**HR (95% CI)**
1st course	GPA 1st course (0–2 vs. 2.5–4)	0.05	0.28 (0.08–1.02)
	Primary tumor controlled at 1st course	0.85	1.13 (0.31–4.08)
	No. of treated metastases at 1st course	0.92	1.02 (0.65–1.61)
	Aggregated tumor volume at 1st course	0.64	0.99 (0.93–1.05)
2nd course	GPA 2nd course (0–2 vs. 2.5–4)	0.15	0.67 (0.39–1.16)
	Primary tumor controlled at 2nd course	**0.02**	0.24 (0.07–0.80)
	No. of treated metastases at 2nd course	0.64	1.07 (0.81–1.40)
	Aggregated tumor volume at 2nd course	0.32	0.91 (0.76–1.10)
	BMV at 2nd course	**0.03**	1.11 (1.01–1.21)

*Values are numbers (percentage) except where noted otherwise*.

*GPA, Graded prognostic assessment score; BMV, Brain metastasis velocity*.

*Bold values indicate the significance level of p < 0.05*.

**Table 4:** Multivariate analysis of correlation to overall survival course-specifically.

	**Variable**	**P**	**HR (95% CI)**
1st course	GPA at 1st course (0–2 vs. 2.5–4)	0.10	0.62 (0.34–1.10)
	Aggregated tumor volume at 1st course	0.40	0.97 (0.91–1.04)
2nd course	GPA at 2nd course (0–2 vs. 2.5–4)	0.76	0.91 (0.51–1.63)
	Aggregated Tumor volume at 2nd course	0.36	0.92 (0.76–1.11)
	BMV at 2nd course	**0.04**	1.10 (1.00–1.21)

*Values are numbers (percentage) except where noted otherwise*.

*GPA, Graded prognostic assessment score; BMV, Brain metastasis velocity*.

*Bold values indicate the significance level of p < 0.05*.

### Toxicity

Within 197 treated lesions 14 sites of RN were observed in 10 (23.8%) patients. None of the patients suffered from severe symptoms. Treatment was only needed for symptomatic RN (*n* = 5, 35.7%). All symptoms resolved after a short course of dexamethasone as temporary treatment and no resection was needed in any of our patients.

**Figure 6:**
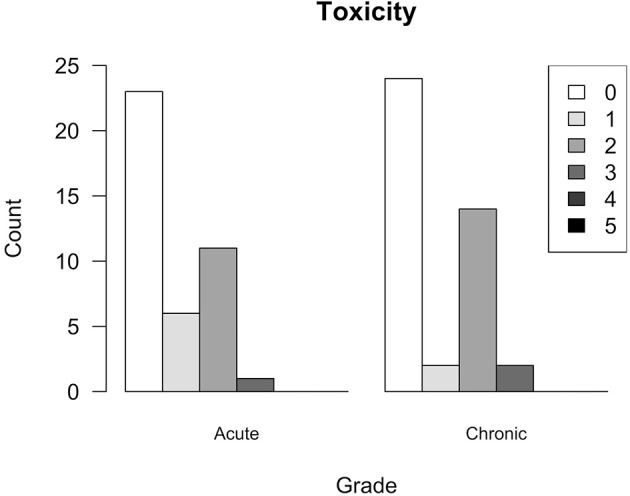
Toxicity. Acute toxicity graded according to Criteria for Adverse Events (CTCAE v4.0) during and ≤ 90 days after respective course of SRS; chronic toxicity occurring >90 days after respective course of SRS. Graph shows highest grade of toxicity observed for individual patients during any course of treatment.

Eighteen of Forty-One (one patient did not have a FU within 90 days) patients (43.9%) developed acute toxicities, with grade 3 in 2 (4.8%), grade 2 in 11 (26.8%), and grade 1 in 6 (14.6%). Eighteen of all Forty-Two patients suffered from low-grade late toxicities: 2 (4.8%) with grade 3, 14 (33.3%) with grade 2, and 2 (4.8%) with grade 1. No grade 4 or 5 toxicity occurred. Figure 6 shows distribution of observed toxicity. Considering presenting symptoms, we observed edema as the most common acute and chronic side effect, provided that edema was diagnosed on regular follow-up MRI scans. It was followed by headache and vertigo, which were defined clinically. Detailed symptoms and grades of acute and late toxicity are reported for patients individually in tables in the Supplementary Material.

## Discussion

Administering SRS only has become the accepted standard of care in treatment of limited number of newly diagnosed brain metastases (2, 4, 6, 9, 12). Compared to WBRT, this approach significantly reduces the risk for neurocognitive impairment (3–5), while at the same time accepting higher rates of distant brain relapse of up to 60% (4, 6, 9, 12). For the frequent scenario of newly occurring brain metastases after initial SRS treatment, a valid standard of care has not been established by prospective trials. WBRT is still the predominantly used treatment option whereas the application of repeated courses of SRS represents an emerging concept, but still provokes safety concerns, since only little data exists.

Recently, a retrospective analysis of 95 patients with 652 metastases treated with repeated SRS only showed that newly diagnosed metastases could be treated safely and effectively with additional courses of SRS (13). Notwithstanding the well-known high rate of distant intracranial progression, only 20% of patients within this study eventually received WBRT as salvage treatment. The authors concluded that WBRT might even be avoided completely for patients with brain metastases. Our findings support this conclusion with a comparable rate of administered WBRT in 17% of our patients.

Although all existing data is limited by the recognized biases of retrospective analyses, patients treated with multiple courses of SRS have acceptable rates of toxicity and no sign of increased neurological death (12, 14, 16–18, 31). Overall, only 14 sites of RN were observed in 197 treated lesions (7%) within our cohort, which is reasonable and comparable to incidences reported in previous studies (14, 16, 30, 32–34). Still, no recommendation exists to what extent repeat radiosurgery is truly safe and what parameters regarding dose overlap should be used to judge about safety. It seems prudent to apply the same general constraints as in first course SRS and to avoid significant dose overlap (e.g., >50% IDL overlap). Also, overall survival is very favorable in our cohort and—although it rather reflects careful patient selection—it is reassuring that deferring WBRT in such patients appears to be a reasonable approach.

It remains controversial which subpopulation of BM patients benefits most from local treatment including repeat SRS (35–37). Determination of predictive factors and development of prognostic indices is crucial in deriving a solid basis for decision-making. The GPA, developed to guide these decisions, has been validated in WBRT as well as SRS treated brain metastases patients (19, 21). Recently, it has been expanded and validated to a disease specific prognostic score (20, 38), even incorporating information of tumor specific mutations (39). Interestingly, as reported by Shultz et al. and Yamamoto et al., GPA remained valid as a prognostic tool for repeat SRS (14, 23). Within our cohort the ds-GPA on univariate and multivariate analysis did not prove to be predictive for overall survival. However, the survival curves split significantly. Interestingly, ds-GPA did not change significantly over time.

Various models assisting clinicians in deciding between administration of WBRT or SRS as initial treatment have been developed. All of them intend to enable estimation of risk of Rodrigues et al. (40) and time to Ayala-Peacock et al. and Press et al. (41, 42) DBF. All of these studies included the number of primarily diagnosed metastases in their scoring system, as it was significantly predictive for early DBF after initial treatment. However, the effect of the estimated number of future metastases or of the dynamics of relapse on overall survival has so far not been investigated. Therefore, an innovative and promising new metric BMV has been developed (24). BMV was established by analysis of 737 patients treated with upfront SRS only for new brain metastases and represents the rate of new metastases that develop over time. The value can be re-calculated over a patient's disease course multiple times and stays prognostic. A multi-institutional study with almost 3,000 has recently validated the BMV as a dominant predictor for OS but the full results have not been published yet (43). BMV has also been included into an announced web-based predictive tool which is not online yet (44). With a median BMV of 3.5 new metastases/year (range: 0.3–22.1) our cohort compared favorably to the reported median BMV of 5.5 (range: 0.2–156.4) by Farris et al. BMV correlated significantly with OS when analyzed as a continuous variable and when factored in the suggested risk stratified groups (BMV < 4, 4–13, >13). Higher BMV predicted for shorter OS, especially when low and high-risk groups were directly compared [*P* = 0.0006, HR (95%CI) = 23.78 (3.88–145.74)] and BMV remained the only significant factor for OS in the multivariable model. Our findings with a median OS of 10 months for the combined intermediate and high-risk group exceed values previously reported by the authors (8.2 and 4.3 months), which might be a combined effect of small sample size and favorable patient selection. BMV seems to be a powerful and promising prognostic metric, even in a small cohort such as ours.

The results of the present study are prone to bias inherent to retrospective studies, particularly selection bias. Furthermore, the ability to analyze patient-specific variables with regard to predictive character is limited due to the small cohort of 42 patients and statistics beyond univariate analysis should be viewed with caution.

## Conclusion

Considering the excellent local control rate, the low toxicity profile and the long OS observed within this study, distant intracranial relapse should not preclude administering SRS: In selected patients with various relapses of brain metastases (DBF) amenable to SRS, repeat courses of SRS can safely be administered to defer or even avoid WBRT. The innovative metric BMV also proved to be prognostic in our cohort and should be further evaluated as a decision-guiding metric.

## Data availability statement

The raw data supporting the conclusions of this manuscript will be made available by the authors, without undue reservation, to any qualified researcher.

## Ethics statement

This study was carried out in accordance with the recommendations of the Humanforschungsgesetz (HFG) and the Kantonale Ethikkommission Zürich with written informed consent from all subjects. All subjects gave written informed consent in accordance with the Declaration of Helsinki. The protocol was approved by the Kantonale Ethikkommission Zürich.

## Author contributions

CF and NA designed and directed the analysis. CF, KB, and SK performed data collection in a database generated by LS, ST-L, and JK. Statistical analysis was performed by CF and KB. CF contributed to the analysis of the results and to the writing of the manuscript. NA and MG supervised the project.

### Conflict of interest statement

The authors declare that the research was conducted in the absence of any commercial or financial relationships that could be construed as a potential conflict of interest.
