# Supplementary material for: Repeated Courses of Radiosurgery for New Brain Metastases to Defer Whole Brain Radiotherapy: Feasibility and Outcome With Validation of the New Prognostic Metric Brain Metastasis Velocity
Source: Front Oncol. 2018 Nov 22;8:551. doi: 10.3389/fonc.2018.00551 (PMC6262082; doi:10.3389/fonc.2018.00551)
Supplement: Supplementary file 1 [file Table_1.DOCX]

Supplementary Material

Repeated courses of radiosurgery for new brain metastases to defer whole brain radiotherapy: feasibility and outcome with validation of the new prognostic metric brain metastasis velocity

C.Fritz^1*^, K. Borsky^1^, L.S. Stark^1^, S. Tanadini-Lang^1^, S.G.C. Kroeze^1^, J. Krayenbühl^1^, M. Guckenberger, N. Andratschke^1^

^11^University Hospital Zurich, Department of Radiation Oncology, Zürich, Switzerland

*** Correspondence:**Corinna Fritz
fricor@posteo.de

# Supplementary Tables

| **Table 1.** Acute toxicity graded from 0-5 per patient and total occurrence | | | | | | |
| --- | --- | --- | --- | --- | --- | --- |
| **Patient** | **Nausea** | **Fatigue** | **Headache** | **Edema** | **Vertigo** | **Other** |
| 1 | **2** | 0 | **3** | 0 | **2** | 0 |
| 2 | 0 | 0 | 0 | 0 | 0 | 0 |
| 3 | 0 | 0 | 0 | 0 | 0 | 0 |
| 4 | 0 | 0 | 0 | 0 | 0 | 0 |
| 5 | 0 | 0 | 0 | 0 | 0 | 0 |
| 6 | **1** | **1** | 0 | **2** | 0 | 0 |
| 7 | 0 | 0 | **2** | **2** | **2** | 0 |
| 8 | 0 | **1** | **2** | **2** | **1** | 0 |
| 9 | 0 | 0 | 0 | **2** | 0 | 0 |
| 10 | 0 | 0 | 0 | 0 | 0 | 0 |
| 11 | 0 | 0 | 0 | 0 | 0 | 0 |
| 12 | 0 | 0 | 0 | 0 | 0 | 0 |
| 13 | 0 | 0 | 0 | 0 | 0 | 0 |
| 14 | 0 | **2** | **3** | **2** | **2** | **2** |
| 15 | 0 | 0 | 0 | 0 | 0 | 0 |
| 16 | 0 | 0 | **2** | **2** | **2** | 0 |
| 17 | 0 | 0 | 0 | 0 | 0 | 0 |
| 18 | 0 | 0 | 0 | 0 | 0 | 0 |
| 19 | 0 | 0 | 0 | 0 | 0 | 0 |
| 20 | 0 | 0 | 0 | 0 | 0 | 0 |
| 21 | 0 | 0 | 0 | **2** | 0 | 0 |
| 22 | 0 | 0 | 0 | **1** | 0 | 0 |
| 23 | / | / | / | / | / | / |
| 24 | 0 | 0 | 0 | 0 | **1** | 0 |
| 25 | 0 | 0 | 0 | 0 | **1** | 0 |
| 26 | 0 | 0 | 0 | 0 | 0 | 0 |
| 27 | 0 | 0 | 0 | 0 | 0 | 0 |
| 28 | 0 | 0 | 0 | **1** | 0 | 0 |
| 29 | 0 | 0 | 0 | **2** | 0 | 0 |
| 30 | **2** | 0 | **2** | 0 | 0 | 0 |
| 31 | 0 | 0 | 0 | 0 | 0 | 0 |
| 32 | 0 | 0 | 0 | 0 | 0 | 0 |
| 33 | 0 | 0 | 0 | 0 | 0 | 0 |
| 34 | 0 | 0 | 0 | 0 | 0 | 0 |
| 35 | 0 | 0 | 0 | 0 | 0 | 0 |
| 36 | 0 | 0 | 0 | 0 | 0 | 0 |
| 37 | 0 | 0 | 0 | 0 | 0 | 0 |
| 38 | 0 | 0 | 0 | **1** | 0 | 0 |
| 39 | 0 | 0 | 0 | 0 | 0 | 0 |
| 40 | 0 | 0 | **2** | **1** | 0 | 0 |
| 41 | 0 | **2** | 0 | **2** | 0 | 0 |
| 42 | 0 | 0 | 0 | **2** | 0 | 0 |
| **Total occurrence** | **3** | **4** | **6** | **13** | **6** | **2** |
| / = no Follow up within the first three months after treatment | | | | | | |

| **Table 2.** Chronic toxicity graded from 0-5 per patient and total occurrence | | | | | | |
| --- | --- | --- | --- | --- | --- | --- |
| **Patient** | **Nausea** | **Fatigue** | **Headache** | **Edema** | **Vertigo** | **Other** |
| 1 | 0 | 0 | 0 | 0 | 0 | 0 |
| 2 | 0 | 0 | 0 | **2** | 0 | 0 |
| 3 | 0 | 0 | 0 | 0 | 0 | 0 |
| 4 | **3** | **2** | **2** | **2** | **2** | **2** |
| 5 | 0 | 0 | 0 | 0 | 0 | 0 |
| 6 | 0 | 0 | 0 | **2** | 0 | 0 |
| 7 | 0 | 0 | 0 | **2** | **2** | 0 |
| 8 | 0 | 0 | **2** | **2** | **1** | 0 |
| 9 | 0 | 0 | 0 | **2** | 0 | 0 |
| 10 | 0 | 0 | 0 | 0 | 0 | 0 |
| 11 | 0 | 0 | 0 | 0 | 0 | 0 |
| 12 | 0 | 0 | 0 | 0 | 0 | 0 |
| 13 | 0 | 0 | 0 | **3** | 0 | 0 |
| 14 | 0 | **1** | **2** | **2** | **2** | 0 |
| 15 | 0 | 0 | 0 | 0 | 0 | 0 |
| 16 | 0 | **1** | **2** | **2** | **2** | 0 |
| 17 | 0 | 0 | 0 | 0 | 0 | 0 |
| 18 | 0 | 0 | 0 | 0 | 0 | 0 |
| 19 | 0 | 0 | 0 | **1** | 0 | 0 |
| 20 | 0 | 0 | 0 | 0 | 0 | 0 |
| 21 | 0 | 0 | 0 | **2** | 0 | 0 |
| 22 | 0 | 0 | 0 | **2** | 0 | 0 |
| 23 | 0 | 0 | 0 | 0 | 0 | 0 |
| 24 | 0 | 0 | 0 | 0 | **1** | 0 |
| 25 | 0 | 0 | 0 | 0 | 0 | 0 |
| 26 | 0 | 0 | 0 | 0 | 0 | 0 |
| 27 | 0 | 0 | 0 | 0 | 0 | 0 |
| 28 | 0 | 0 | 0 | 0 | 0 | 0 |
| 29 | 0 | 0 | 0 | **2** | 0 | 0 |
| 30 | 0 | 0 | 0 | 0 | 0 | 0 |
| 31 | 0 | 0 | 0 | **2** | 0 | 0 |
| 32 | 0 | 0 | 0 | 0 | 0 | 0 |
| 33 | 0 | 0 | 0 | **2** | 0 | 0 |
| 34 | 0 | 0 | 0 | **2** | 0 | 0 |
| 35 | 0 | 0 | 0 | 0 | 0 | 0 |
| 36 | 0 | 0 | 0 | 0 | 0 | 0 |
| 37 | 0 | 0 | 0 | 0 | 0 | 0 |
| 38 | 0 | 0 | 0 | 0 | 0 | 0 |
| 39 | 0 | 0 | 0 | 0 | 0 | 0 |
| 40 | 0 | 0 | 0 | 0 | 0 | 0 |
| 41 | 0 | 0 | 0 | 0 | 0 | 0 |
| 42 | 0 | **2** | **2** | **2** | **1** | 0 |
| **Total occurrence** | **1** | **4** | **5** | **17** | **7** | **1** |
